# Molecular targeting of the Aurora-A/SMAD5 oncogenic axis restores chemosensitivity in human breast cancer cells

**DOI:** 10.18632/oncotarget.20610

**Published:** 2017-09-01

**Authors:** Mateusz Opyrchal, Malgorzata Gil, Jeffrey L. Salisbury, Mathew P. Goetz, Vera Suman, Amy Degnim, James McCubrey, Tufia Haddad, Ianko Iankov, Chenye B. Kurokawa, Nicole Shumacher, James N. Ingle, Evanthia Galanis, Antonino B. D’Assoro

**Affiliations:** ^1^ Department of Medical Oncology, Mayo Clinic College Of Medicine, Rochester, MN, USA; ^2^ Department of Biochemistry and Molecular Biology, Mayo Clinic College Of Medicine, Rochester, MN, USA; ^3^ Department of Molecular Medicine, Mayo Clinic College Of Medicine, Rochester, MN, USA; ^4^ Department of Microbiology and Immunology, East Carolina University, Greenville, NC, USA; ^5^ Department of Medicine, Roswell Park Cancer Institute, Buffalo, NY, USA

**Keywords:** breast cancer, chemoresistance, tumor progrression, EMT, stemness

## Abstract

Although the majority of breast cancers initially respond to the cytotoxic effects of chemotherapeutic agents, most breast cancer patients experience tumor relapse and ultimately die because of drug resistance. Breast cancer cells undergoing epithelial to mesenchymal transition (EMT) acquire a CD44^+^/CD24^-^/ALDH1^+^ cancer stem cell-like phenotype characterized by an increased capacity for tumor self-renewal, intrinsic drug resistance and high proclivity to develop distant metastases. We uncovered in human breast tumor xenografts a novel non-mitotic role of Aurora-A kinase in promoting breast cancer metastases through activation of EMT and expansion of breast tumor initiating cells (BTICs). In this study we characterized the role of the Aurora-A/SMAD5 oncogenic axis in the induction of chemoresistance. Breast cancer cells overexpressing Aurora-A showed resistance to conventional chemotherapeutic agents, while treatment with alisertib, a selective Aurora-A kinase inhibitor, restored chemosensitivity. Significantly, SMAD5 expression was required to induce chemoresistance and maintain a breast cancer stem cell-like phenotype, indicating that the Aurora-A/SMAD5 oncogenic axis promotes chemoresistance through activation of stemness signaling. Taken together, these findings identified a novel mechanism of drug resistance through aberrant activation of the non-canonical Aurora-A/SMAD5 oncogenic axis in breast cancer.

## INTRODUCTION

Tumor relapse associated with distant metastasis is a major cause of death for breast cancer patients [1]. The major hindrances in eradicating metastatic lesions include high tumor cell heterogeneity, self-renewal, and intrinsic resistance to chemotherapeutic agents [2, 3]. While 70% of human breast carcinomas fall into luminal CD44^-^/CD24^+^ subtypes, which are estrogen receptor alpha (ERa) positive and are initially sensitive to endocrine therapy [4], triple negative breast carcinomas (TNBCs) show a basal-like CD44^+^/CD24^-^ phenotype and low/absent levels of expression of ERα, progesterone receptor (PR) and HER2 tyrosine kinase receptor [5]. ERα and HER2-targeted therapies are ineffective for TNBC [6]. Conventional chemotherapy is the only effective therapeutic option, and TNBCs generally respond well to cytotoxic agents in the earlier stages of the disease [7]. Unfortunately, metastatic ER+ and TNBCs will eventually exhibit resistance to anti-cancer drugs leading to tumor progression and poor outcomes [8-10]. Therefore, a better understanding of the molecular mechanisms responsible for drug resistance is imperative to accelerate the development of innovative strategies to restore chemosensitivity and improve the progression-free and overall survival of breast cancer patients.

Several studies have demonstrated that activation of epithelial to mesenchymal transition (EMT) reprogramming promotes drug resistance and tumor progression [11]. EMT is a key biological process observed in embryonic development, tissue regeneration and organ fibrosis during which cells progressively lose the epithelial features and gain mesenchymal properties [12]. Epithelial cancer cells can also activate EMT reprogramming that drives the progressive loss of adhesion molecules (E-cadherin and claudin) and concurrent increased expression of mesenchymal proteins (N-cadherin and vimentin) that favor cell motility and invasion [13-15]. Breast cancer cells that undergo EMT acquire a basal-like CD44^+^/CD24^-^ stemness phenotype characterized by a greater capacity for tumor self-renewal and early onset of distant metastasis [16-19]. The discovery that breast tumors contain a sub-population of cells with stemness properties, termed breast tumor initiating cells (BTICs), is critical to understanding the molecular mechanisms responsible for therapeutic failures and poor patient outcomes in advanced breast cancer [20, 21]. BTICs generally exhibit intrinsic resistance to conventional chemotherapy through different mechanisms that involve aberrant ABC transporter expression, increased ALDH1 activity, enhanced DNA repair activity to genotoxic stress and activation of self-renewal and survival pathways [22]. Breast tumors treated with conventional anti-cancer drugs may have residual drug-resistant BTICs, and it is these cells that promote tumor re-growth, metastatic dissemination and poor clinical outcomes.

EMT and stemness reprogramming are induced by several mechanisms: oncogenic pathways such as MAPK, Wnt, NOTCH, PI3K/AKT/mTOR and TGFβ/SMADs; components of the extracellular matrix such as collagen and hyaluronic acid; and hostile conditions such as hypoxia [23]. Prior studies have shown the Aurora-A mitotic kinase as a critical mediator of EMT and stemness in cancer cells [24, 25]. In agreement with these findings, we have demonstrated in MCF-7 breast cancer xenografts engineered to express a constitutively active Raf-1 oncoprotein, the non-mitotic role of Aurora-A kinase in promoting distant metastasis through activation of EMT and development of CD44^+^/CD24^-^ BTICs [26]. Moreover, we have discovered the role of activated Aurora-A kinase in the induction of phosphorylation of the SMAD5 transcription factor that plays a critical role in orchestrating EMT and stemness reprogramming. Although the function of aberrant Aurora-A kinase activity in the development of cancer cell resistance to chemotherapeutic agents has been established [27-29], the underlying molecular mechanisms responsible for this have not been determined.

Here we define the role of the Aurora-A kinase in the induction of chemoresistance of breast cancer cells through phosphorylation and activation of SMAD5. We demonstrate that SMAD5 expression is required to induce a CD44^+^/CD24^-^/ALDH1^+^ breast cancer stem cell-like phenotype, suggesting that the Aurora-A/SMAD5 axis promotes chemoresistance through activation of stemness signalling in breast cancer. Taken together, these findings reveal a novel mechanism of drug resistance and provide the preclinical rationale to developstrategies that target the non-canonical Aurora-A/SMAD5 oncogenic axis and restore chemosensitivity in breast cancer cells.

## RESULTS

### Raf-1-induced chemoresistance is not linked to loss of p53 function

**Figure 1:**
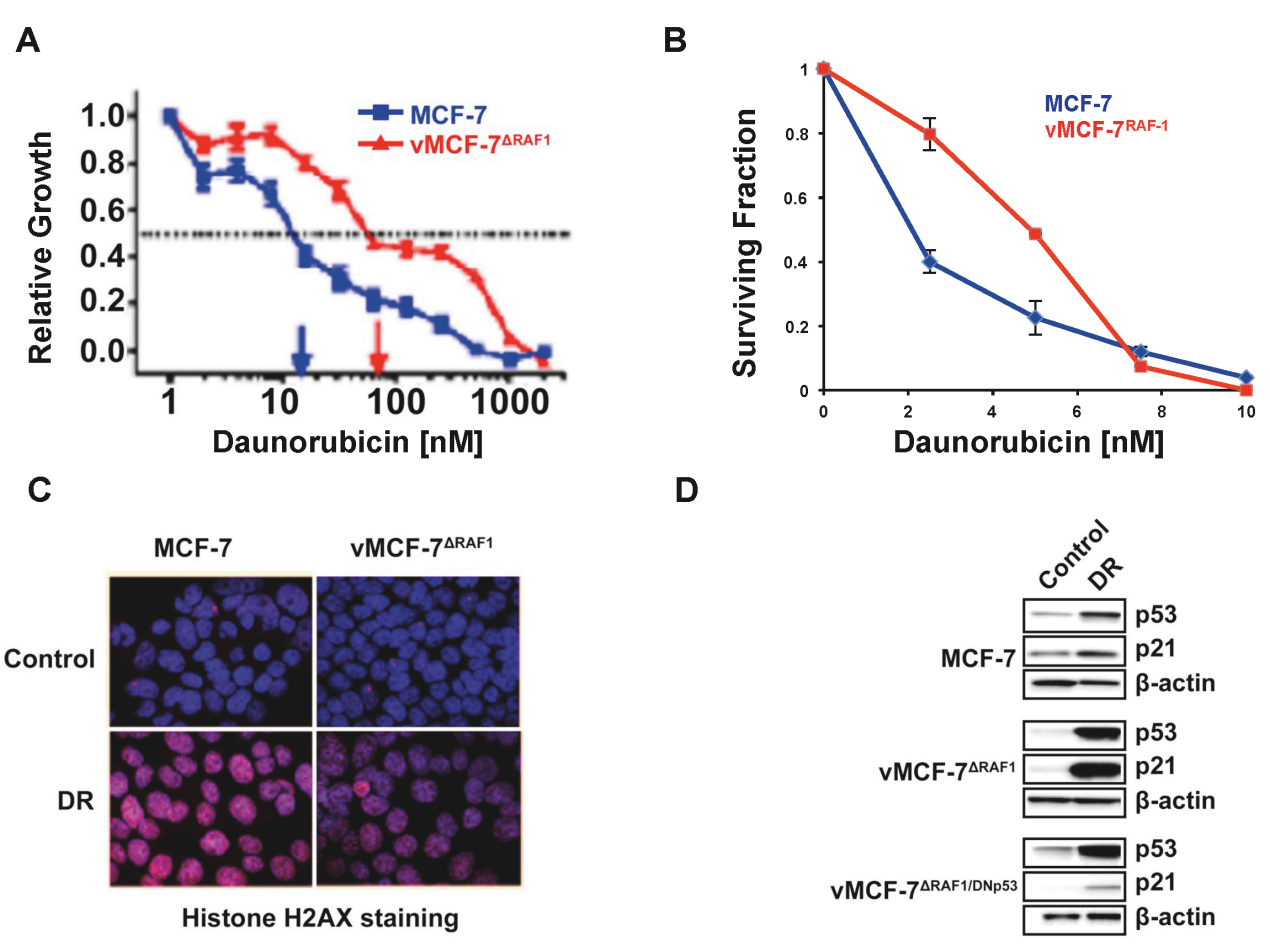
Development of Chemoresistance in Breast Cancer Cells **A.** Cell Proliferation Assay showing that vMCF-7^∆Raf1^ cells exhibit higher resistance to escalating doses of DR compared to parental MCF-7^pZipNeo^ cells. **B.** Clonogenic assay showing that vMCF-7^∆Raf1^ cells are more resistant to DR compared to parental MCF-7^pZipNeo^ cells. **C.** Immunofluorescence assay showing decreased histone H2AX staining in vMCF-7^∆Raf1^ cells compared to parental MCF-7^pZipNeo^ cells following DR-induced genotoxic stress. Histone H2AX was labeled in red and nuclei were labeled in blue with Hoechst stain. **D.** Immunoblot assay showing expression of p53 and p21 before and following DR-induced genotoxic stress in MCF-7^pZipNeo^, vMCF-7^∆Raf1^ and vMCF-7^∆Raf1/DNp53^ cells.

We have demonstrated that overexpression of a constitutive active Raf-1 mutant in ERa+ MCF-7 breast cancer cells (vMCF-7^∆Raf1^) resulted in constitutive activation of MAPK oncogenic signaling and enhanced tumorigenic properties *in vivo* [26, 30]. Because aberrant activation of MAPK signaling is associated with chemoresistance [31], we investigated whether vMCF-7^∆Raf1^ cells exhibited resistance to daunorubicin (DR), a genotoxic stress-inducing agent [32]. Parental MCF-7 and vMCF-7^∆Raf1^ cells were treated with increased concentration of DR and relative cell proliferation was analyzed after 7 days. vMCF-7^∆Raf1^ cells exhibited increased resistance to DR treatment when compared to MCF-7 cells (Figure 1A). A clonogenic assay corroborated the increased resistance of vMCF-7^∆Raf1^ cells to DR compared to parental MCF-7 cells (Figure 1B). The reduced chemosensitivity was also associated with lower levels of DR-induced genotoxic stress as confirmed by decreased histone H2AX staining in vMCF-7^∆Raf1^ cells (Figure 1C). For the reason that resistance to genotoxic anti-cancer agents has been functionally linked to loss of p53 activity [33], we investigated whether Raf-1-induced chemoresistance was linked to impairment of p53 function. Expression of p53 and its downstream transcriptional target p21 was increased following DR treatment in both MCF-7 and vMCF-7^∆Raf1^ cells (Figure 1D). These results demonstrate that vMCF-7^∆Raf1^ cells have an intact p53/p21 tumor suppressor axis and increased chemoresistance of vMCF-7^∆Raf1^ cells is not linked to loss of p53 activity.

To determine the extent to which p21 expression was strictly dependent on p53 activity and not induced by Raf-1 signaling after genotoxic stress, we engineered vMCF-7^∆Raf1^ cells to overexpress a dominant negative (DN) p53^val135^ mutant to mask the function of wild-type p53 (vMCF-7^∆Raf1/DNp53^) [32, 34]. Previous studies have shown that cancer cells with DNp53^val135^ mutant also exhibit increased resistance to anti-cancer drugs [35]. vMCF-7^∆Raf1/DNp53^ cells showed low levels of p21 before and after genotoxic stress, indicating that p21 expression is dependent on wild-type p53 activity (Figure 1D). Because intact p53/p21 axis prevents onset of centrosome amplification after genotoxic stress [31], we determined the percentage of cancer cells harboring centriole overduplication following DR treatment. MCF-7 and vMCF-7^∆Raf1^ cells showed lack of centriole overduplication following genotoxic stress, corroborating the integrity of p53/p21 axis in vMCF-7^∆Raf1^ cells (Supplementary Figure 1). Taken together, these findings demonstrate that Raf-1-induced chemoresistance does not require loss of p53 function in MCF-7 cells.

### Aurora-A kinase enhances Raf-1-induced chemoresistance

**Figure 2:**
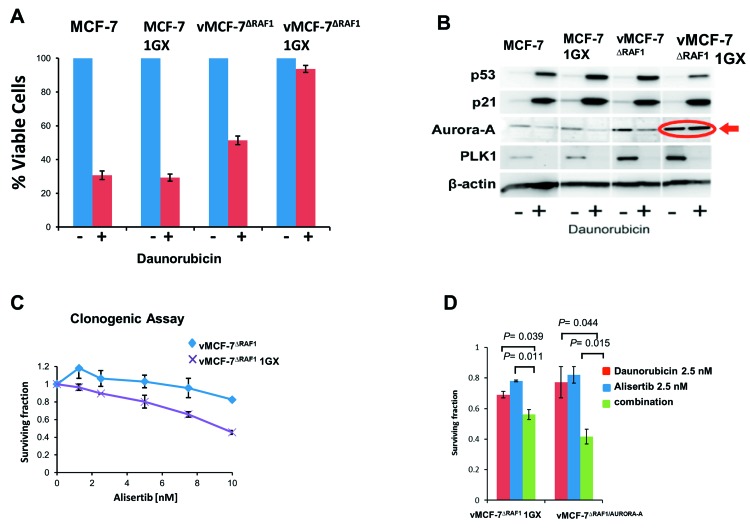
Aurora-A Kinase Activity is Required to Induce Chemoresistance **A.** MTT assay showing that vMCF-7^∆Raf1^ 1GX cells display the highest resistance to DR compared to parental cells. Graph represents the average from three independent experiments. **B.** Immunoblot assay showing expression of p53, p21, Aurora-A and PLK1 before and after DR-induced genotoxic stress in variant and parental MCF-7 cells. **C.** Clonogenic assay showing that treatment with the Aurora-A kinase inhibitor alisertib reduces the survival of vMCF-7^∆Raf1^ and vMCF-7^∆Raf1^ 1GX cells. **D.** Clonogenic assay showing that treatment with alisertib restores sensitivity to DR in vMCF-7^∆Raf1^ 1GX and vMCF-7^∆Raf1/Aurora-A^ cells. Results are presented as the average of three independent experiments *± SEM.*

We have previously demonstrated that *ex-vivo* vMCF-7^∆Raf1^ cells (first generation xenografts, 1GX) established from metastatic vMCF-7^∆Raf1^ tumor xenografts, exhibited overexpression of Aurora-A mitotic kinase, while *ex-vivo* MCF-7 1GX cells established from non-metastatic MCF-7 tumor xenografts (used as control) maintained nominal levels of Aurora-A [26]. Aurora-A overexpression in vMCF-7^∆Raf1^ 1GX cells was functionally linked to EMT and the genesis of BTICs with a CD44^+^/CD24^Low/-^ basal-like phenotype [26]. Based on these findings, we investigated the role of Aurora-A kinase activity in enhancing Raf-1-induced chemoresistance. MCF-7, MCF-7 1GX, vMCF-7^∆Raf1^ and vMCF-7^∆Raf1^ 1GX cells were treated with DR and cell viability was assessed after 7 days by MTT assay (Figure 2A). MCF-7 and MCF-7 1GX cells showed sensitivity to DR treatment as both cell populations exhibited more than 60% decrease in cell proliferation after genotoxic stress. In contrast, vMCF-7^∆Raf1^ cells displayed 50% reduction in cell viability and vMCF-7^∆Raf1^ 1GX cells showed the highest resistance, i.e., 90%, to DR. A clonogenic assay also corroborated the increased resistance of vMCF-7^∆Raf1^ 1GX cells to DR compared to parental MCF-7 and vMCF-7^∆Raf1^ cells (Supplementary Figure 2). Next, we established the extent to which high chemoresistance of vMCF-7^∆Raf1^ 1GX cells was dependent on loss of p53 function that may have occurred during *in vivo* growth of vMCF-7^∆Raf1^ cells. Following induction of genotoxic stress, vMCF-7^∆Raf1^ 1GX cells exhibited increased expression of p53 and p21 in a similar fashion of parental cells (Figure 2B). These results demonstrate that high chemoresistance observed in vMCF-7^∆Raf1^ 1GX cells is not linked to loss of p53/p21 axis.

To establish whether deregulated expression of Aurora-A was linked to high chemoresistance, we analyzed Aurora-A levels before and after genotoxic stress in vMCF-7^∆Raf1^ 1GX and parental cells. Significantly, only vMCF-7^∆Raf1^ 1GX cells didn’t show down-regulation of Aurora-A after DR treatment (Figure 2B). Conversely, both vMCF-7^∆Raf1^ 1GX and parental cells that received DR treatment exhibited down-regulation of another mitotic kinase, Polo-like kinase 1 (PLK1) that plays a central role in tumorigenesis (Figure 2B) [36]. A clonogenic assay showed that vMCF-7^∆Raf1^ 1GX cells are more sensitive to alisertib (a selective Aurora-A kinase inhibitor) than parental vMCF-7^∆Raf1^ cells (Figure 2C), suggesting that vMCF-7^∆Raf1^ 1GX cells are dependent on Aurora-A kinase activity for their growth and survival. Moreover, to assess the role of Aurora-A in enhancing chemoresistance, we performed a clonogenic assay using vMCF-7^∆Raf1^ 1GX (with high endogenous levels of Aurora-A) and vMCF-7^∆Raf1^ cells engineered to overexpress Aurora-A (vMCF-7^∆Raf1/Aurora-A^) as previously demonstrated [26]. Combination of DR with alisertib restored sensitivity to DR in vMCF-7^∆Raf1^ 1GX and vMCF-7^∆Raf1^ cells (Figure 2D), demonstrating that Aurora-A kinase activity is able to induce DR resistance.

**Figure 3:**
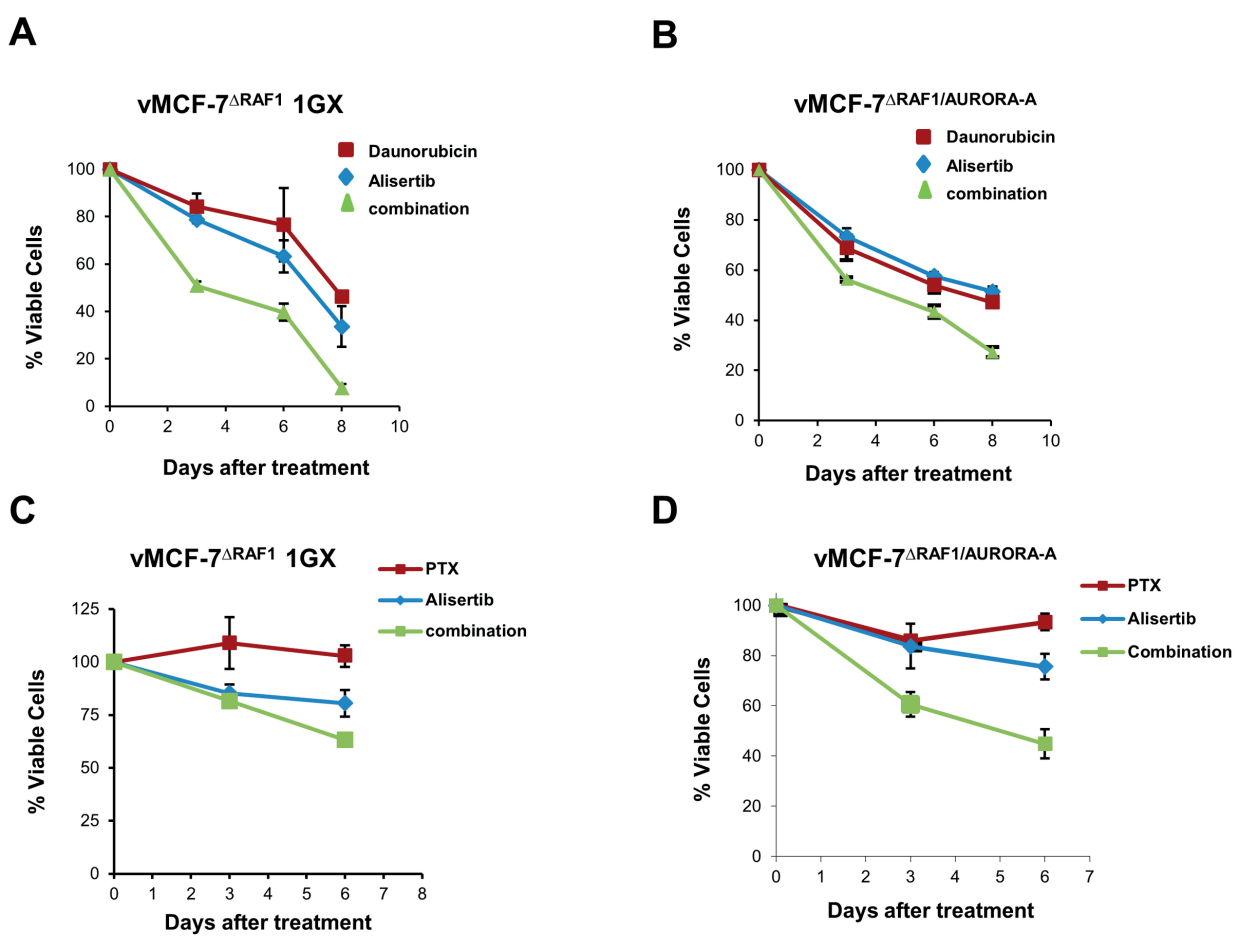
Aurora-A-Targeted Therapy Restores Chemosensitivity **A.**-**D.** MTT assay showing that treatment with the Aurora-A kinase inhibitor alisertib restores sensitivity to DR and PTX in vMCF-7^∆Raf1^ 1GX and vMCF-7^∆Raf1/Aurora-A^ cells. Results are presented as the average of three independent experiments *± SEM.*

Next, we performed a MTT assay to validate the role of Aurora-A kinase in inducing resistance to DR and paclitaxel (PTX), a conventional chemotherapeutic agent that stabilizes microtubules and triggers mitotic arrest and apoptosis [37]. vMCF-7^∆Raf1^ 1GX and vMCF-7^∆Raf1/Aurora-A^ cells were treated with ½ IC50 alisertib alone and in combination with ½ IC50 DR or ½ IC50 PTX (Figure 3). IC50s for alisertib, DR and PTX were previously established (Supplementary Figure 3). Treatment of vMCF-7^∆Raf1^ 1GX and vMCF-7^∆Raf1^ cells with DR or PXT in combination with alisertib restored chemosensitivity (Figure 3A-3D). Taken together, these results demonstrate that inhibition of Aurora-A kinase activity restores sensitivity to conventional chemotherapeutic agents in drug-resistant breast cancer cells.

### Pharmacological inhibition of Aurora-A kinase activity reduces phosphorylated SMAD5 and restores chemosensitivity

**Figure 4:**
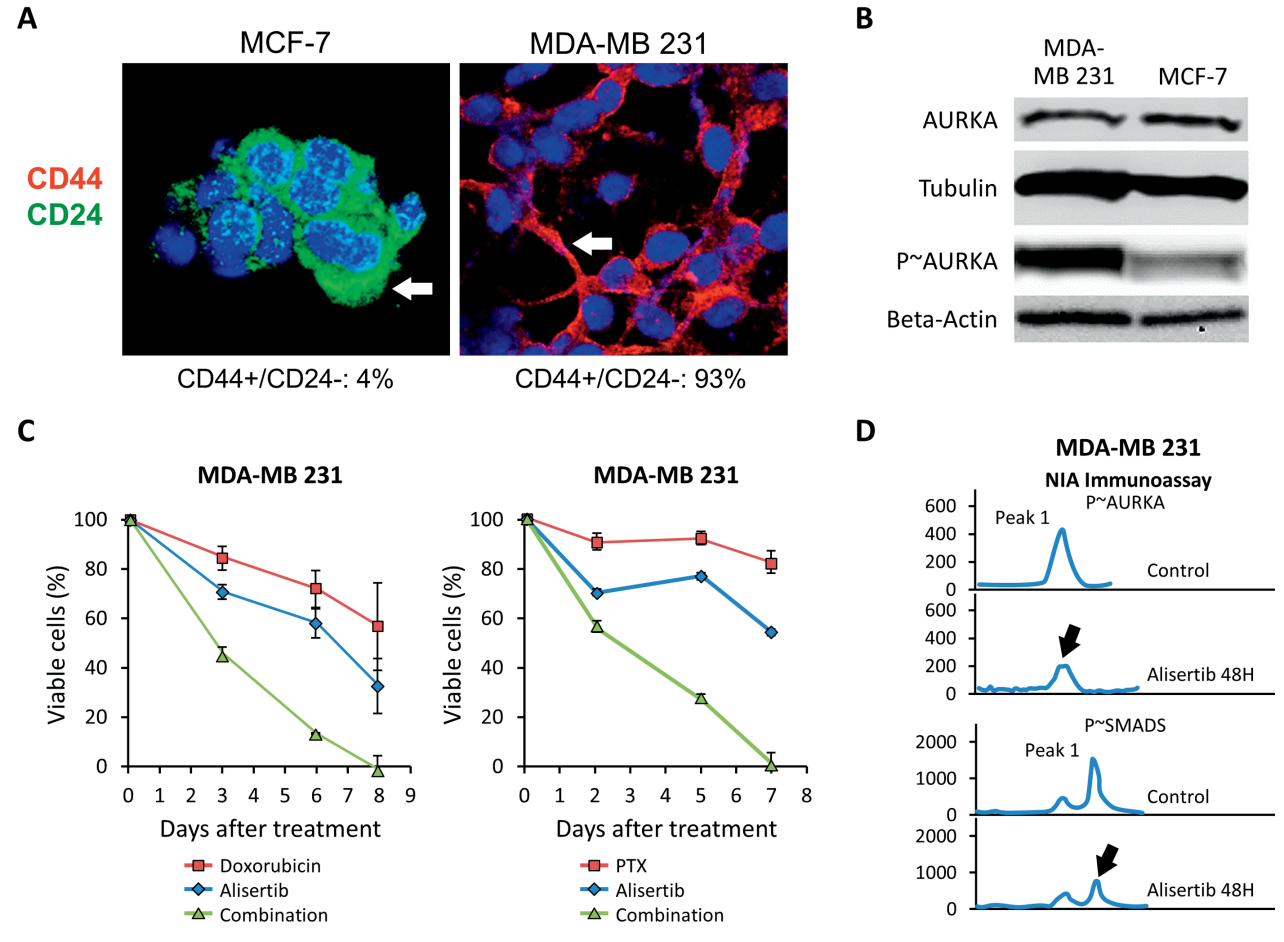
Pharmacologic Targeting of Aurora-A Restores Chemosensitivity **A.** Immunofluorescence analysis showing the percentage of CD44^+^/CD24^-^ sub-fraction in MCF-7 and MDA-MB 231 breast cancer cells. **B.** Immunoblot assay showing the expression of total and phosphorylated (p∼) Aurora-A in MDA-MB 231 and MCF-7 cells. Tubulin and Beta-actin expression were used as loading control. **C.** MTT assay showing that treatment with alisertib restores sensitivity to Doxorubicin and PTX in MDA-MB 231 cells. Results are presented as the average of three independent experiments *± SEM.* **D.** Highly sensitive NIA immunoassay showing a decrease in Aurora-A and SMAD5 phosphorylation after treatment with alisertib in MDA-MB 231 Cells. Results are derived from three independent experiments with comparable outcomes.

To corroborate the role of Aurora-A in enhancing chemoresistance in TNBC, we employed the highly metastatic MDA-MB 231 cell line [38]. MDA-MB 231 cells showed a basal-like CD44^+^/CD24^-^ phenotype that was linked to high endogenous levels of phosphorylated Aurora-A (p∼Aurora-A) compared to MCF-7 cells that exhibited a luminal CD44^-^/CD24^+^ phenotype and nominal levels of p∼Aurora-A (Figure 4A and 4B). These results are in agreement with our previous findings demonstrating that overexpression of p∼Aurora-A is restricted to the basal-like CD44^+^/CD24^-^ sub-fraction in breast tumors [26]. Therefore, we investigated the role of Aurora-A kinase activity in promoting resistance to doxorubicin (an antracycline with comparable activity of daunorubicin that is used in TNBC treatment) and PTX. Treatment of MDA-MB 231 cells with ½ IC50 doxorubicin or ½ IC50 PXT had nominal effects on cell viability (Figure 4C). Significantly, when MDA-MB 231 cells were treated with ½ IC50 Alisertib in combination with the same concentration of the chemotherapeutic agent, we observed a significant decrease in cellular proliferation compared to cells treated with doxorubicin or PTX alone (Figure 4C). IC50s for alisertib, DR and PTX in MDA-MB 231 cells had been previously established (Supplementary Figure 4).

Since we previously demonstrated that Aurora-A kinase activity is required to induce nuclear phosphorylation and activation of SMAD5 transcription factor in vMCF-7^∆Raf1^ cells [26, 39], we assessed the role of Aurora-A kinase activity in inducing SMAD5 phosphorylation in MDA-MB 231 cells. Inhibition of Aurora-A phosphorylation was associated with reduced p∼SMAD5, corroborating the role of Aurora-A kinase activity in promoting SMAD5 phosphorylation (Figure 4D). Taken together, these findings validate, in TNBC cells, the critical role of Aurora-A kinase activity in inducing chemoresistance. Likewise, they also suggest that aberrant Aurora-A kinase activity may favor a chemoresistant phenotype through activation of SMAD5.

### SMAD5 expression is required to induce chemoresistance

**Figure 5:**
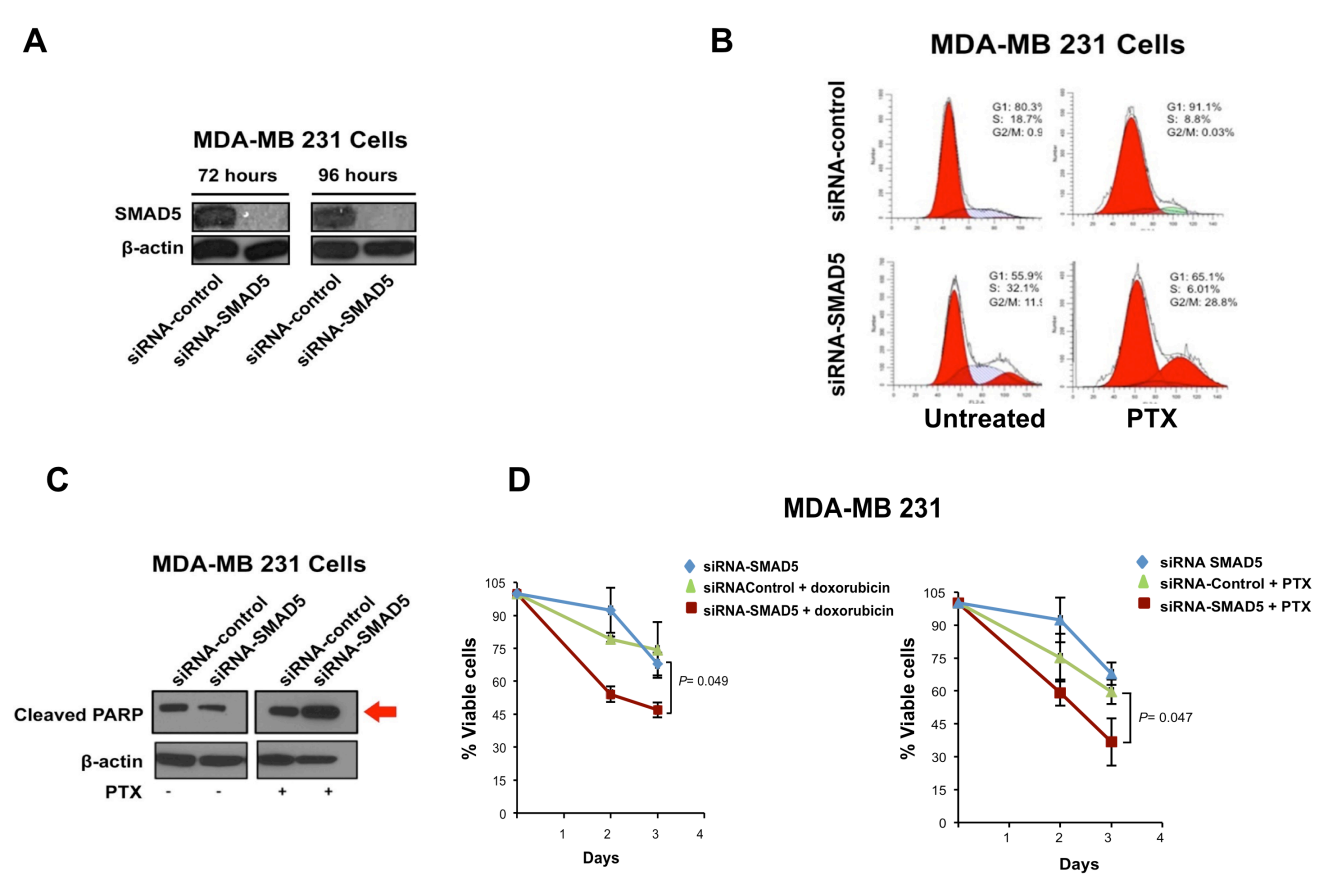
SMAD5 Expression is Required to Induce Chemoresistance **A.** Immunoblot assay showing reduced SMAD5 expression in MDA-MB 231 cells after treatment with siRNA-SMAD5 for 72 and 96 hours. **B.** FACS analysis showing that treatment with siRNA-SMAD5 for 96 hours restores PTX-induced G2/M phase arrest in MDA-MB 231 cells. Scramble siRNAs were used as control. Results are derived from three independent experiments with comparable outcomes. **C.** Immunoblot assay showing expression of cleaved-PARP in MDA-MB 231 cells treated for 96 hours with PTX and/or siRNA-SMAD5. Scramble siRNAs were used as control. **D.** MTT assay showing that treatment with siRNA-SMAD5 restores sensitivity to Doxorubicin and PTX in MDA-MB 231 cells. Scramble siRNA were used as control. Results are presented as the average of three independent experiments *± SEM.*

To explore the function of SMAD5 in inducing chemoresistance, we employed SMAD5 specific siRNAs to knockdown SMAD5 expression in MDA-MB 231 cells (Figure 5A). Because treatment with PTX induces G2/M cell cycle arrest in chemosensitive cancer cells [40], MDA-MB 231 cells were treated with PTX and/or siRNAs targeting SMAD5 (Scramble siRNAs were used as control) and cell cycle profile was analyzed by FACS assay. While treatment with PTX did not induce G2/M cell cycle arrest, treatment with PTX and siRNA-SMAD5 resulted in G2/M cell cycle arrest, indicating that siRNA-SMAD5 restored PTX anti-mitotic activity (Figure 5B). Because PTX-induced cell cycle arrest is functionally linked to activation of apoptosis [40], we tested the levels of cleaved-PARP (a marker of apoptosis) in MDA-MB 231 cells treated with PTX and/or siRNA-SMAD5 (Scramble siRNAs were used as control). Increased expression of cleaved-PARP was observed only in cells treated with PTX and siRNA-SMAD5 (Figure 5C). Next, we performed a MTT assay to explore the role of SMAD5 expression in inducing chemoresistance. MDA-MB 231 cells were treated with siRNA-SMAD5 alone and in combination with ½ IC50 doxorubicin or ½ IC50 PTX. Treatment of MDA-MB 231 cells with doxorubicin or PXT in combination with siRNA-SMAD5 restored chemosensitivity (Figure 5D). Combination of siRNA-SMAD5 with ½ IC50 doxorubicin or ½ IC50 PTX also restored chemosensitivity in BT549 TNBC cells (Supplementary Figure 5). Taken together, these results demonstrate that SMAD5 expression is required to induce chemoresistance in TNBC cells that exhibit high endogenous Aurora-A kinase activity.

### SMAD5-induced chemoresistance is linked to maintenance of a CD44^+^/CD24^-^/PROCR^+^ cancer stem-like Phenotype

**Figure 6:**
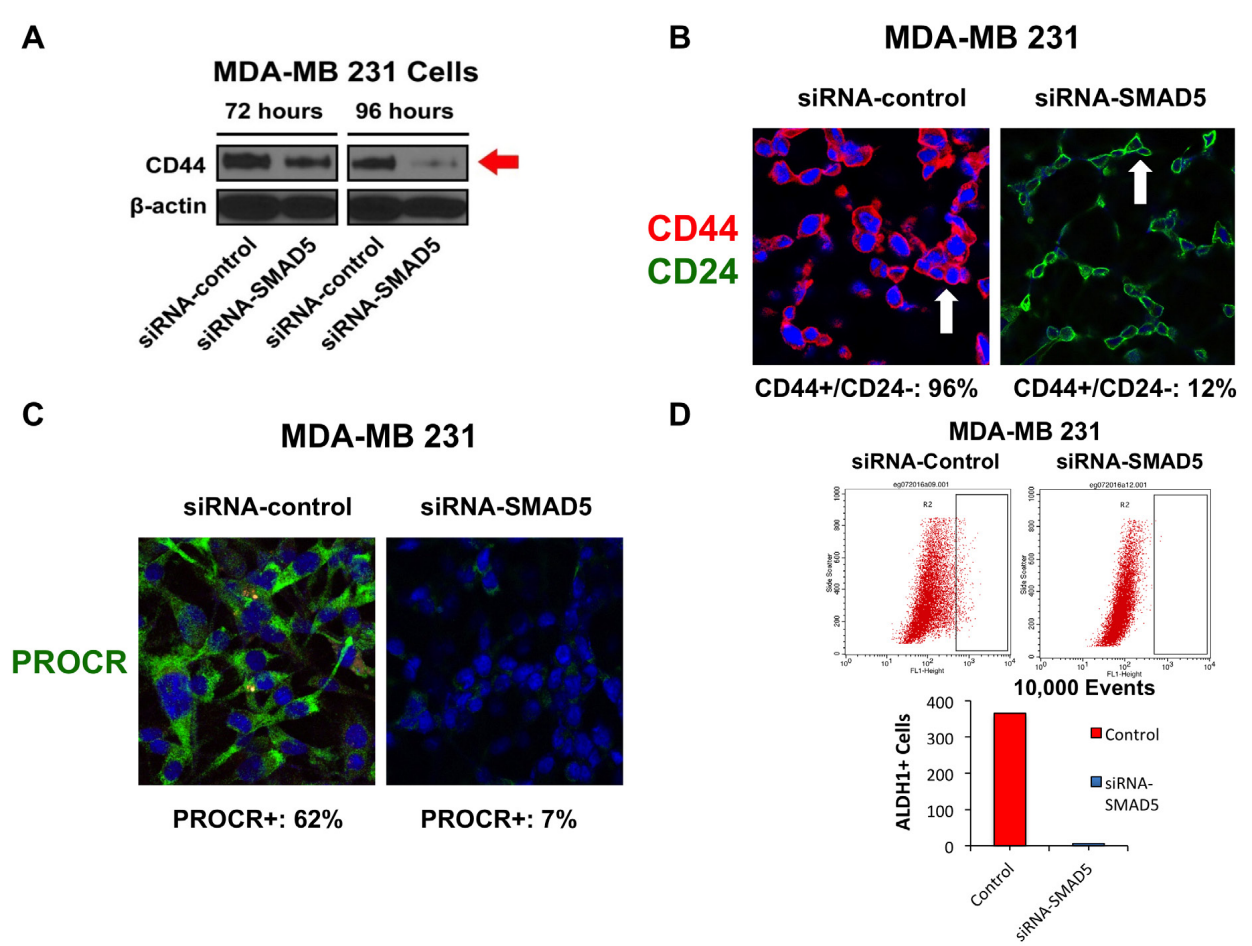
**A.** Immunoblot assay showing the expression of CD44 stemness marker after treatment with siRNA-SMAD5 for 72 and 96 hours in MDA-MB 231 cells. Scramble siRNA were used as control. **B.** Immunofluorescence assay showing the percentage of CD44^+^/CD24^-^ sub-fraction in MDA-MB 231 cells before and after treatment with siRNA-SMAD5 for 96 hours. Scramble siRNA were used as control. **C.** Immunofluorescence assay showing the percentage of PROCR^+^ MDA-MB 231 cells before and after treatment with siRNA-SMAD5 for 96 hours. Scramble siRNA were used as control. **D.** FACS showing ALDH1 activity in MDA-MB 231 cells before and after treatment with siRNA-SMAD5 for 96 hours. Scramble siRNA were used as control. Graph showing the number of ALDH1+ cells (average from three independent experiments) in MDA-MB 231 cells before and after treatment with siRNA-SMAD5 for 96 hours. Scramble siRNA were used as control. Experiments were performed in triplicate with comparable results.

Because expression of the CD44 stemness marker promotes chemoresistance in breast cancer cells [41], we investigated the extent to which SMAD5 activity was required to induce CD44 expression in MDA-MB 231 cells. Following treatment of MDA-MB 231 cells with siRNA-control or siRNA-SMAD5, CD44 expression was markedly reduced only in MDA-MB 231 cells treated with siRNA-SMAD5 (Figure 6A). Moreover, treatment with siRNA-SMAD5 induced a luminal CD44^-^/CD24^+^ phenotype in MDA-MB 231 cells (Figure 6B), indicating that SMAD5 expression is required to maintain a CD44^+^/CD24^-^ cancer stem cell-like phenotype. Although CD44 and CD24 are well-established markers of breast cancer stemness, they are not universal surface markers for the definitive characterization of a breast cancer stem-like phenotype. For this reason, we also investigated the expression of PROCR, a well-established breast cancer stemness marker linked to higher tumorigenicity [16]. The majority of MDA-MB 231 cells treated with siRNA-control were positive for PROCR staining, while MDA-MB 231 cells treated with siRNA-SMAD5 showed a marked decrease of PROCR expression (Figure 6C). Finally, to determine the extent to which SMAD5 expression was required to induce ALDH1 activity that is responsible for stemness capacity and high chemoresistance, we performed an Aldeofluor assay in MDA-MB 231 cells treated with siRNA-control or siRNA-SMAD5. ALDH1 activity was significantly reduced in MDA-MB 231 cells treated with siRNA-SMAD5 (Figure 6D), corroborating the findings that SMAD5 expression is required to promote tumor stemness and chemoresistance.

## DISCUSSION

Acquired and intrinsic resistance to anti-cancer drugs still remains a major issue that limits the effectiveness of therapy and the survival of breast cancer patients [42]. Several studies have shown that a sub-fraction of breast cancer cells termed BTICs undergo EMT reprogramming and typically exhibit a basal-like CD44^+^/CD24^-^ phenotype harboring cancer stem-like features [43-45]. BTICs are usually refractory to conventional anti-cancer drugs through their capacity for increased DNA repair, overexpression of ABC-transporters, high ALDH1 activity and inhibition of apoptosis pathways [22]. The intrinsic drug resistant activity of BTICs offers an explanation for tumor re-growth and progression following treatment with current endocrine and chemotherapy regimens. Nonetheless, the molecular mechanisms linking activation of stemness signaling with intrinsic resistance toward anti-cancer drugs are poorly understood.

In this study, we defined the role of the Aurora-A/SMAD5 oncogenic axis in the induction of chemoresistance in breast cancer cells. First, we employed luminal ER+ MCF-7 cells with constitutively active Raf/MAPK signaling (vMCF-7^∆Raf1^) that exhibit a more aggressive phenotype than parental cells [26]. vMCF-7^∆Raf1^ cells showed higher resistance to the genotoxic agent DR compared to MCF-7 cells, corroborating the causal role of Raf/MAPK oncogenic signaling in promoting resistance to conventional anti-cancer drugs [46]. Although it has been showed that Raf/MAPK signaling induces expression of multi-drug resistant P-glycoprotein [47], the molecular mechanisms responsible for Raf/MAPK-mediated drug resistance are poorly characterized. Because impaired p53 function is linked to chemoresistance in cancer cells [48], we investigated whether constitutive activation of Raf/MAPK signaling reduced the expression of p53 and its downstream target p21 after genotoxic stress. Significantly, Raf/MAPK-driven chemoresistance was not linked to loss of integrity of p53/p21 tumor suppressor axis, suggesting that Raf/MAPK oncogenic signaling induces drug resistance through p53-independent mechanisms in MCF-7 cells.

Since we have established a novel oncogenic cross-talk between Raf/MAPK signaling and Aurora-A kinase in the activation of EMT and breast cancer progression [26], we aimed to determine the role of Aurora-A in promoting Raf/MAPK-induced chemoresistance. High endogenous levels of Aurora-A in *ex-vivo* vMCF-7^∆Raf1^ 1GX cells were linked to resistance to conventional chemotherapeutic agents. Moreover, Aurora-A expression levels remained high after DR-induced genotoxic stress in vMCF-7^∆Raf1^ 1GX cells that exhibited the highest resistance to DR compared to parental cells regardless the presence of an intact p53/p21 axis. Significantly, pharmacologic targeting of Aurora-A kinase activity with alisertib restored chemosensitivity, demonstrating that Aurora-A activity is required to promote Raf/MAPK-induced chemoresistance in ER+ breast cancer cells. Although one of the mechanisms by which Aurora-A induces oncogenic transformation is through down-regulation of p53 activity [49], our findings demonstrate that Aurora-A-induced resistance to genotoxic agents does not require abrogation of p53 function. One of the alternative molecular mechanisms by which aberrant Aurora-A kinase activity may induce chemoresistance in cancer cells is likely through activation of EMT and stemness reprogramming. In agreement with this hypothesis, we have demonstrated the causative role of Aurora-A in promoting the expansion of CD44^+^/CD24^-^/ER^-^ BTICs responsible for the onset of distant metastases in vMCF-7^∆Raf1^ tumor xenografts [26]. To corroborate the function of Aurora-A in promoting Raf/MAPK-induced chemoresistance, we employed highly metastatic MDA-MB 231 TNBC cells with elevated endogenous Raf/MAPK activity [50]. MDA-MB 231 cells showed a basal-like CD44^+^/CD24^-^ phenotype that was linked to high levels of p∼Aurora-A and resistance to doxorubicin and PTX. Following treatment with alisertib, MDA-MB 231 cells gained sensitivity to chemotherapeutic agents, demonstrating the role of Aurora-A kinase activity in inducing chemoresistance also in TNBC cells. In agreement with our previous results [26], Aurora-A-induced drug resistance was linked to phosphorylation of SMAD5, a key transcription factor downstream of TGF-b/BMP signaling involved in tumor progression [51, 52]. Significantly, molecular targeting of SMAD5 expression was linked to inhibition of a CD44^+^/CD24^-^/PROCR^+^ cancer stem cell-like phenotype and low ALDH1 activity. Taken together, these findings demonstrate that SMAD5 expression is required to induce chemoresistance through maintenance of tumor stemness in TNBC cells.

**Figure 7:**
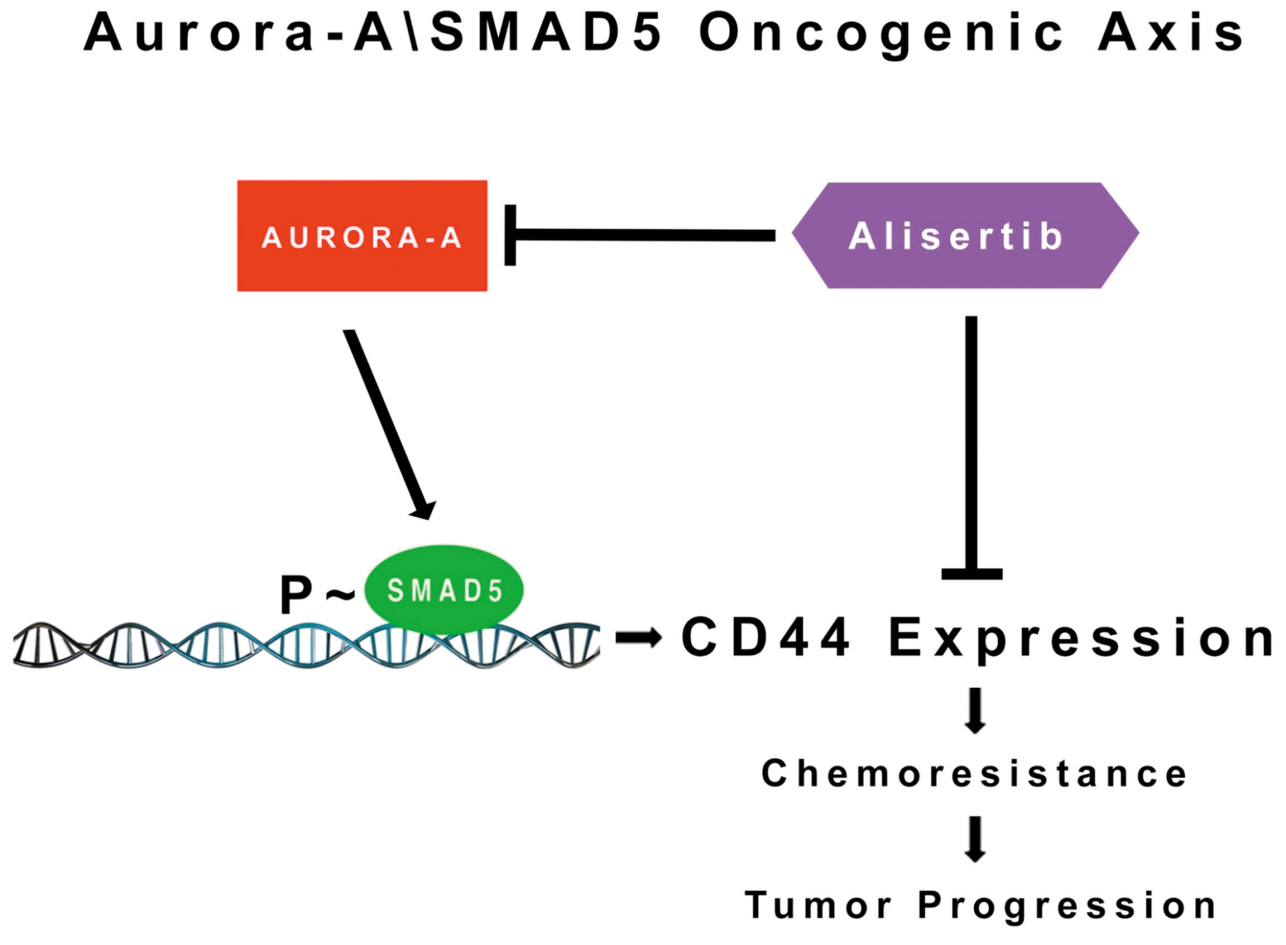
Molecular Targeting of the Aurora-A/SMAD5 Oncogenic Axis Restores Chemosensitivity: Aberrant Aurora-A kinase activity induces phosphorylation of SMAD5 transcriptional factor that in turn will promote expression of CD44 receptor and activation of stemness signaling responsible for chemoresistance and tumor progression Pharmacologic targeting of Aurora-A kinase activity can be effective to restore chemosensitivity through inhibition of the Aurora-A/SMAD5 oncogenic axis and stemness signalings in cancer cells.

In conclusion, the study presented here proposes a novel mechanism of breast cancer progression by which aberrant activation of the Aurora-A/SMAD5 oncogenic axis induces expression of CD44 and PROCR receptors and increased ALDH1 activity that are critical to maintain a cancer stem cell-like phenotype responsible for chemoresistance and tumor progression (Figure 7). Conversely, pharmacologic targeting of Aurora-A restores chemosensitivity through inhibition of SMAD5 transcriptional activity leading to restoration of a more differentiated luminal CD44^-^/CD24^+^/PROCR^-^ phenotype with low ALDH1 activity and impairment of tumor stemness. The combination of conventional chemotherapeutic drugs with inhibitors of Aurora-A kinase activity represents a promising therapeutic strategy for the management of chemoresistant breast cancer, particularly TNBCs that currently lack FDA-approved targeted therapies.

## MATERIALS AND METHODS

### Human breast cancer cell lines

The human breast cancer cell lines MCF-7 and MDA-MB 231 were obtained from ATCC (Manassas, Virginia, USA). Variant MCF-7 expressing the constitutive active Raf-1 and DN p53^val135^ mutants, vMCF-7^∆Raf1^ 1GX and MCF-7 1GX cells were generated as previously described [26, 32, 34]. All cell lines were maintained in DMEM medium containing 5mM glutamine, 1% penicillin/streptomycin and 10% FBS at 37 C in 5% CO2 atmosphere.

### Cell proliferation assay

Cancer cells were seeded in 96-well plates at a density of 100 cells/well in the absence or presence of escalating doses of DR. After 7 days incubation, cells were recovered with trypsin/EDTA (Life Technologies, Inc.), stained with trypan blue and counted with a hemacytometer. Experiments were performed in quadruplicate and *± SEM* was calculated.

### Clonogenic assay

Cells were plated in duplicates on 6 well plates with densities varying from 50 to 200 cells/well. The cells were treated with daunorubicin, paclitaxel or alisertib at indicated concentrations and cultured in a 37°C, 5% CO_2_ incubator for 12 days. The media were replaced every 3 days. The cells were fixed with methanol/acetic acid (7:1) and stained with 0.5% crystal violet in methanol for 60 min. Individual colonies of more than 50 cells were counted. The survival fraction was calculated as follows: Surviving fraction = colonies counted/(cells seeded × PE) x100) where PE is the plating efficiency that represents the ratio of the number of colonies to the number of cells seeded.

### Immunoblot, immunofluorescence and FACS assays

Immunoblot, Immunofluorescence and FACS assays were performed as previously described [26]. Antibodies employed to perform these studies were the followings: p53 (D07 DAKO), p21 (Oncogene), Centrin 20h5 (Mayo Clinic, Rochester, Minnesota, USA), H2AX, Aurora-A, p∼Aurora-A, SMAD5, PLK1, cleaved-PARP, CD44 (Cell Signaling, Danvers, Massachusetts, USA), CD44, CD24 (BD Pharmigen, San Jose, California, USA), PROCR and Beta-actin (Sigma, St. Louis, Missouri, USA). Results are derived from three independent experiments with comparable outcomes.

### Nano-fluidic immunoassay (NIA)

Lysates were analyzed using a NanoPro 1000 system (ProteinSimple Inc.) with an optimized protocol. Primary antibodies were used at 1:50 dilution for 2 hours. Antibodies employed to perform the NIA assay were the followings: p∼Aurora-A (Cell Signaling, Danvers, Massachusetts, USA) and p∼SMAD5 (Abcam, Cambridge, Massachusetts, USA). Results are derived from three independent experiments with comparable outcomes.

### Chemoresistance studies

Cell viability was determined employing the MTT [3-(4, 5-dimethylthiazol-2-yl)-2, 5-diphenyltetrazolium] colorimetric assay (ATCC, Manassas, Virginia, USA). Cancer Cells were plated at a density of 10^4^ cells/well in 96-well plates. Twenty-four hours after seeding, the cells were treated with paclitaxel and/or alisertib. Cell viability assay was performed at indicated time points after treatment initiation following the manufacturer’s instructions. Briefly, cells were incubated with 10% cell proliferation kit I added directly to the medium for 4 hours at 37 °C, followed by cell lysis with a detergent reagent (ATCC) overnight in the dark at room temperature. Absorbance was determined in a SpectraMax microplate reader (Molecular Devices, Sunnyvale, CA) at 570 nm in three to six different wells per group and results were calculated as the percent of optical density in the treated wells versus the untreated (used as control). Results are presented as the means of three independent experiments *± SEM.*

### RNA interference assay

The siGENOME Human SMAD5 siRNAs and siGENOME Non-targeting siRNA (Thermo Scientific, West Palm Beach, Florida, USA) were transfected into the breast cancer cells at the final concentration 25nM using DharmaFECT 1 Transfection Agent (Thermo Scientific) according to the manufacturer’s instructions. The siRNA-treated cells were collected after 72 and 96 hours and lysed with cell lysis buffer (Cell Signaling, Danvers, Massachusetts, USA) to assess SMAD5 expression.

### Cell cycle analysis

Trypsinized and floating cells were pooled, washed with PBS-EDTA, and fixed in 70% (v/v) ethanol. For the assessment of DNA contents, cell were stained with PI and monitored by FACSCalibur. Cell cycle distribution was determined with the ModFit LT program (Verity Software House Inc.). Results are derived from three independent experiments with comparable outcomes.

### ALDH1 activity assay

MDA-MB 231 cells were treated with siRNA-control or siRNA-SMAD5 for 96 hours and ALDH1 activity was detected using the Aldeofluor assay kit (STEMCELL Technologies, Canada) according to the manufacturer’s instructions. Results are derived from three independent experiments with comparable outcomes.

## SUPPLEMENTARY MATERIAL FIGURES


