# Supplementary material for: Molecular targeting of the Aurora-A/SMAD5 oncogenic axis restores chemosensitivity in human breast cancer cells
Source: Oncotarget. 2017 Sep 1;8(53):91803–16. doi: 10.18632/oncotarget.20610 (PMC5710966; doi:10.18632/oncotarget.20610)
Supplement: Supplementary file 1 [file oncotarget-08-91803-s001.pdf]

Molecular targeting of the Aurora-A/SMAD5 oncogenic axis restores chemosensitivity in human breast cancer cells

SUPPLEMENTARY MATERIALS

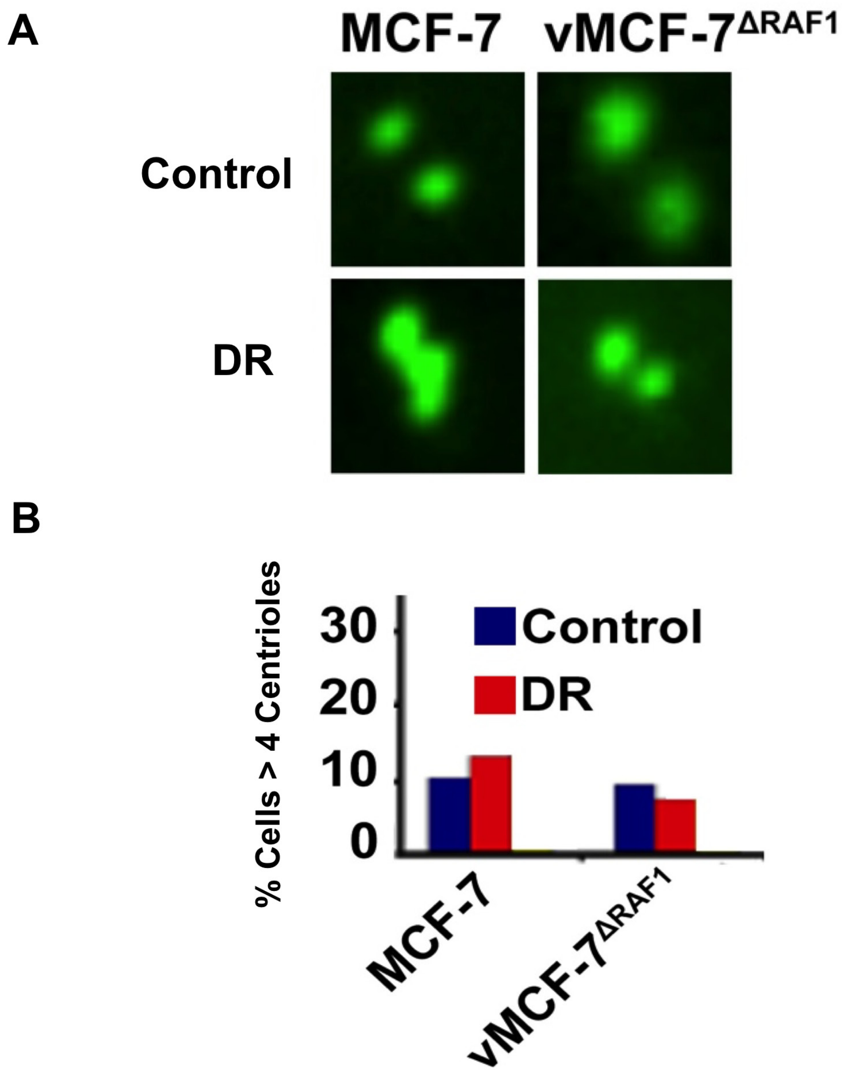

Supplementary Figure 1: Analysis of Centriole Duplication Before and After Genotoxic Stress: Immunofluorescence assay showing representative images of centrioles before and after DR-induced genotoxic stress in MCF-7 and vMCF-7$^{\Delta Raf1}$ cells. Centrioles were labeled in green with 20h5 centrin antibody. Graph showing the average of % cells with more than 4 centrioles before and after DR-induced genotoxic stress from three independent experiments.

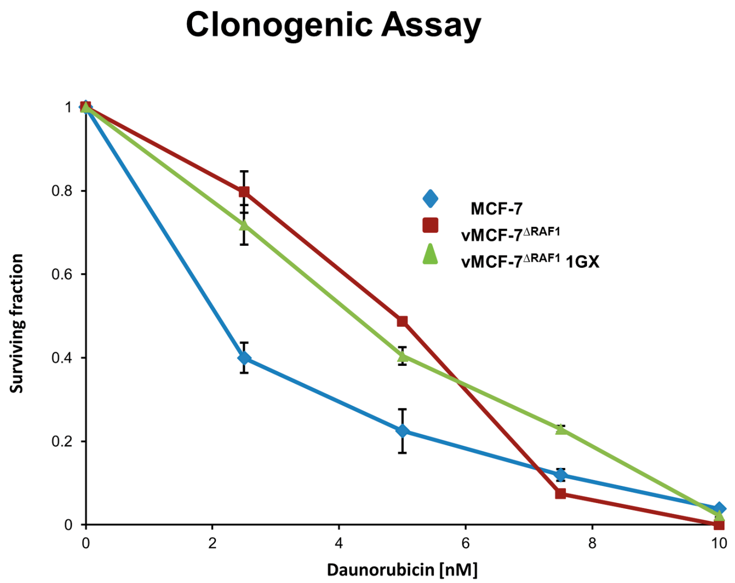

Supplementary Figure 2: Clonogenic Assay Following Induction of Genotoxic Stress: Clonogenic assay showing that vMCF-$^{7\Delta Raf1}$ and vMCF-$^{7\Delta Raf1}$ 1GX cells are more resistant to DR compared to parental MCF-^{7pZipNeo} cells.

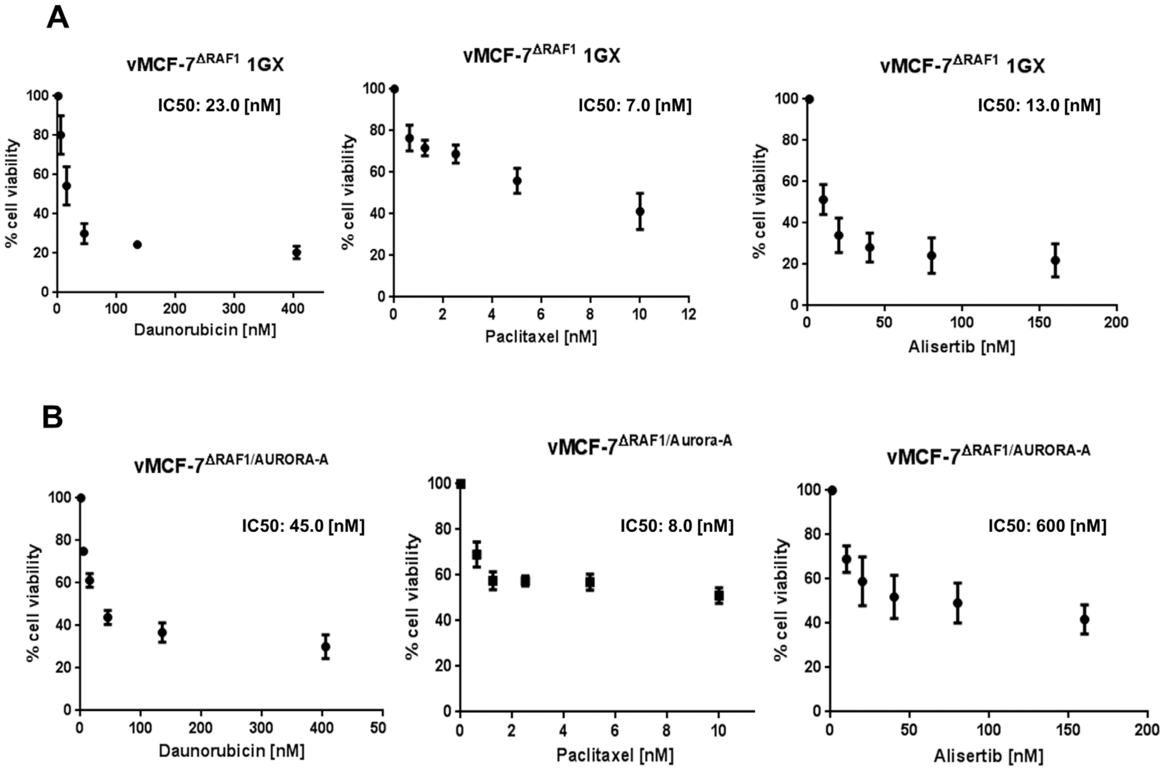

Supplementary Figure 3: Survival of Breast Cancer Cells: (A) MTT Assay was performed using vMCF-$^{7\Delta Raf1}$ 1GX cells to establish the IC50 for DR, PTX and alisertib. (B) MTT Assay was performed using vMCF-$^{7\Delta Raf1/Aurora\text{-}A}$ cells to establish the IC50 for DR, PTX and alisertib.

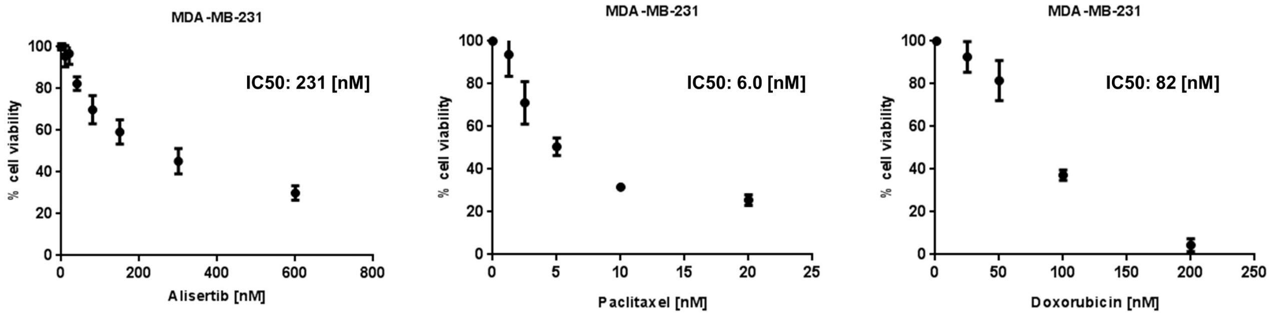

Supplementary Figure 4: Survival of MDA-MB 231 TNBC Cells: MTT Assay was performed using MDA-MB 231 cells to establish the IC50 for doxorubicin, PTX and alisertib.

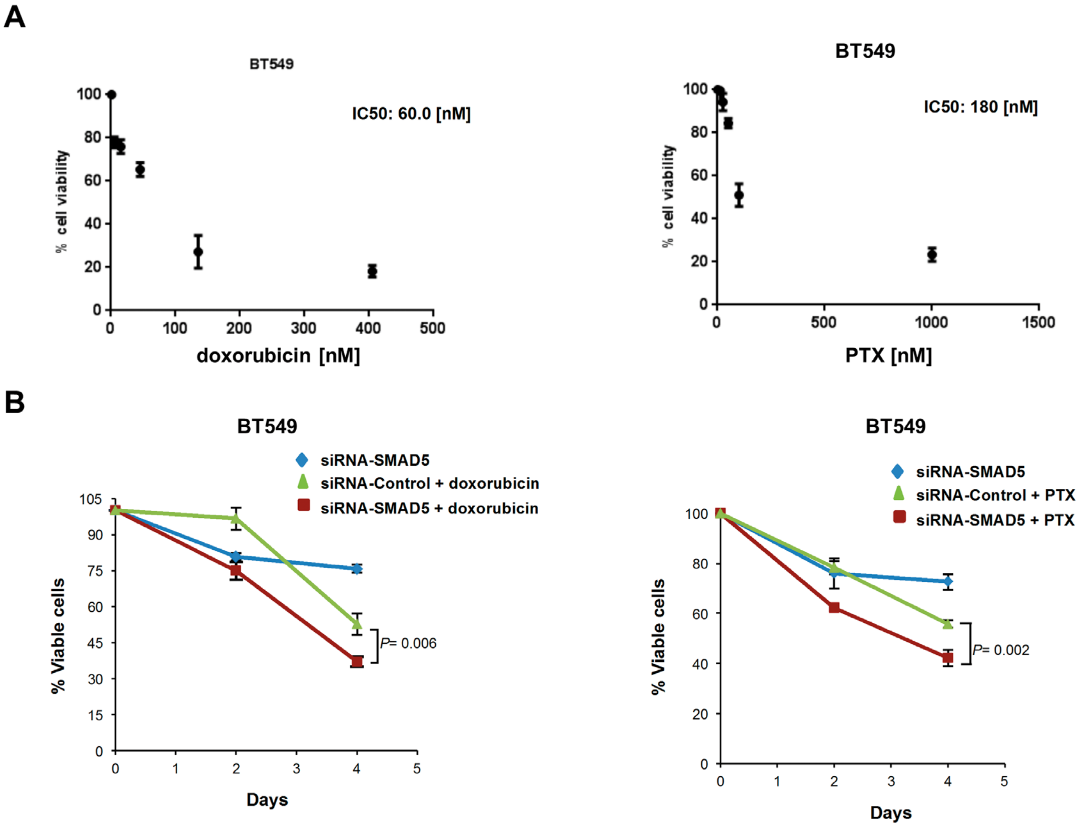

Supplementary Figure 5: Survival of BT549 TNBC Cells: (A) MTT Assay was performed using BT549 cells to establish the IC50 for doxorubicin and PTX. (B) MTT assay showing that treatment with siRNA-SMAD5 restores sensitivity to Doxorubicin and PTX in BT549 cells. Scramble siRNA were used as control. Results are presented as the average of three independent experiments ± *SEM*.
